# The Implementation of a Complication Avoidance Care Bundle Significantly Reduces Adverse Surgical Outcomes in Orthopedic Trauma Patients

**DOI:** 10.3390/jcm9124006

**Published:** 2020-12-11

**Authors:** Thomas Lustenberger, Simon Lars Meier, René Danilo Verboket, Philipp Störmann, Maren Janko, Johannes Frank, Ingo Marzi

**Affiliations:** Department of Trauma, Hand and Reconstructive Surgery, Hospital of the Johann Wolfgang Goethe-University Frankfurt am Main, 60590 Frankfurt am Main, Germany; simon.meier@kgu.de (S.L.M.); Rene.Verboket@kgu.de (R.D.V.); philipp.stoermann@kgu.de (P.S.); maren.janko@kgu.de (M.J.); Johannes.Frank@kgu.de (J.F.); ingo.marzi@kgu.de (I.M.)

**Keywords:** adverse events, complications, surgery, prevention, bundle

## Abstract

Background: Surgical complications are associated with a significant burden to patients and hospitals and are increasingly discussed in recent literature. This cohort study reviewed surgery-related complications in a Level I trauma center. The effect of a complication avoidance care bundle on the rate of surgical complications was analyzed. Methods: All complications (surgical and nonsurgical) that occur in our trauma department are prospectively captured using a standardized documentation form and are discussed and analyzed in a weekly trauma Morbidity and Mortality (M&M) conference. Surgical complication rates are calculated using the annual surgical procedure numbers. Based on discussions in the M&M conference, a complication avoidance care bundle consisting of five measures was established: (1) Improving team situational awareness; (2) reducing operating room traffic by staff members and limiting door-opening events; (3) preoperative screening for infectious foci; (4) adapted preoperative antibiotic prophylaxis in anatomic regions with a high risk of infectious complications; and (5) use of iodine-impregnated adhesive drape. Results: The number of surgical procedures steadily increased over the study years, from 3587 in 2015 to 3962 in 2019 (an increase of 10.5%). Within this 5-year study period, the overall rate of surgical complications was 0.8%. Surgical site infections were the most frequently found complications (*n* = 40, 24.8% of all surgical complications), followed by screw malposition (*n* = 20, 12.4%), postoperative dislocations of arthroplasties (*n* = 18, 11.2%), and suboptimal fracture reduction (*n* = 18, 11.2%). Following implementation of the complication avoidance care bundle, the overall rate of surgical complications significantly decreased, from 1.14% in the year 2016 to 0.56% in the study year 2019, which represents a reduction of 51% within a 3-year time period. Conclusions: A multimodal strategy targeted at reducing the surgical complication rate can be successfully established based on a transparent discussion of adverse surgical outcomes. The combination of the different preventive measures was associated with reducing the overall complication rate by half within a 3-year time period.

## 1. Introduction

Complications following surgical procedures have received increasing attention in recent years. A systematic review showed that almost two-thirds of in-hospital events are associated with surgical care [1]. As a consequence, numerous interventions have been proposed to increase patient safety, including relegating specific procedures to high-volume centers, establishing training programs for different surgical procedures, and improving the quality of teamwork in operating rooms [2,3]. In addition, a number of surgical checklists have been developed and, most importantly, protocols to prevent “wrong site, wrong procedure, and wrong person” surgery [4,5,6,7,8]. These checklists include different components: a pre-procedure verification process, in which the entire team ensures that all relevant documents, information, and equipment are available; marking the operative site; and performing a “time out” before the procedure [9]. However, complications such as surgical site infections (SSI) and technical errors during the procedure itself, leading to unfavorable implant position or intraoperative lesions of nerve or vascular structures, are also surgical adverse events, having high impact on the patient’s outcome. Therefore, prevention of surgical complications remains of utmost importance.

Scoring tools, such as the Clavien–Dindo scale system or the Comprehensive Complication Index (CCI) have been developed to provide clinically meaningful data on the severity of the complications [10,11]. For trauma patients, the Clavien–Dindo complication scoring system has even been adapted to grade the severity of post-traumatic complications in patients managed surgically and nonsurgically [12]. These scoring systems are increasingly used as nonmortality endpoints for research purposes, but also for quality improvement measures such as Morbidity and Mortality (M&M) conferences. These M&M conferences are traditionally considered as ideal forums to discuss complications that have occurred and to explore the management details of each particular case. In carefully reviewing the records and specifics of the adverse event, the primary goal is to revisit errors and complications to gain more insight without blame. Thus, the M&M conferences create an immense opportunity to learn from medical errors, complications, and unanticipated outcomes. They are an ideal meeting where teaching can be promoted and surgeons can learn from each other’s complications [13,14]. However, numerous manuscripts have elucidated that participants of M&M conferences often feel that the purpose of the discussion is more to assign blame for an error than to improve patient safety [15,16]. Nevertheless, honest discussion of adverse outcomes in a trustful atmosphere is a fundamental requirement to identify system-based problems and to improve patient care and safety.

The goal of the present study was threefold: (1) to review surgical complications documented in a Level I trauma center, (2) to develop a bundle of different complication prevention measures based on the weekly case-related M&M conference discussions, and (3) to analyze the impact of this combination of different measures on the overall rate of surgical complications.

## 2. Material and Methods

This cohort study was performed at the University Hospital Frankfurt/Main, department of Trauma, Hand and Reconstructive surgery, which is a Level I, supraregional maximum-care trauma center. The study period included January 2015 to December 2019. All patients receiving surgical procedures during this time period were included, and the total number of operations was calculated. Patients not undergoing surgery were excluded from further analysis.

All in-hospital complications (surgical and nonsurgical, pre-, intra-, and postoperative) of the department are prospectively captured using a standardized documentation form, as previously described by Wagner et al. [17]. In brief, this complication recording sheet includes demographic parameters and information on comorbidities of the patient, as well as information on the diagnosis, surgical procedure, and the complication registered. All complications are discussed weekly and analyzed in an intradepartmental M&M conference, and solutions to prevent repetition of these adverse events are developed in an intercollegial discussion.

Complications are categorized as surgical vs. nonsurgical events. Surgical complications include all perioperative complications associated with the surgical procedure itself, mainly comprising SSI, postoperative dislocation after arthroplasty, screw malposition, suboptimal fracture reduction, and intraoperative lesions of vascular or nerve structures. Surgical site infections are defined according to the Centers for Disease Control and Prevention (CDC), including incisional SSI (superficial and deep) and organ/space SSI [18]. Screw malposition is defined as any intra-articular or intraspinal position of a screw. Pedicle screws affecting the spinal cord, nerve roots, great vessels, pleura, esophagus, and trachea are likewise considered as malpositioned. Suboptimal fracture reduction and/or fixation is defined as any indication for revision. This is based on postoperative radiological images, which are reviewed daily in an interdisciplinary way by trauma surgeons and radiologists.

Nonsurgical events include complications not directly associated with the surgery, such as hospital-acquired pneumonia or urinary tract infection.

### 2.1. Complication Avoidance Care Bundle

Based on the documented complications in the years 2015 and 2016, a specific complication avoidance care bundle was gradually developed during the weekly M&M conference. Ultimately, this bundle included the following 5 measures:

#### 2.1.1. Improving Team Situational Awareness

To improve the team situational awareness, a structured team time out (TTO) was seen as a central key to putting the entire surgical and anesthesiological team on the same information level at the beginning of the surgical procedure. The TTO, performed prior to skin incision, includes—among other points—the introduction of the entire team and the respective level of experience, discussion of specific stages of the surgical procedure, and completeness of surgical equipment and hardware. Furthermore, any specific comments can be raised by any of the team members. An improved situational awareness at an individual, but also team level, was intended to reduce the rate of suboptimal surgical results such as insufficient fracture reduction, and screw or plate malposition, but also intraoperative lesions of significant vessels or nerves.

#### 2.1.2. Reducing Operating Room Traffic by Staff Members and Limiting Door-Opening Events

During the entire procedure, the operating room (OR) doors remained closed and were immediately closed after door opening, thereby minimizing suboptimal airflow events and reducing the noise level in the operating theatre. All staff members were routinely instructed to comply with this measure.

#### 2.1.3. Preoperative Screening for Infectious Foci (Including Urinary Tract Infection)

Prior to emergent hip surgeries, a screening for bacteriuria was routinely undertaken in the emergency department with urine cultures as part of the routine preoperative laboratory testing. Furthermore, a screening for methicillin-resistant Staphylococcus aureus (MRSA) was likewise performed directly on hospital admission. For patients undergoing nonurgent surgery, screening was done in the trauma outpatient clinic.

#### 2.1.4. Adapted Preoperative Antibiotic Prophylaxis in Anatomic Regions with a High Risk of Infectious Complications

The routine perioperative antibiotic prophylaxis includes cefuroxime 1.5 g iv, administered at least 30 min prior to skin incision and with a redosing interval of 4 h. In high-risk anatomic regions with regard to SSI, including the hip and shoulder with their proximity to the groin and axilla, respectively, Metronidazol 1 g iv was added to additionally cover anaerobic pathogens.

#### 2.1.5. Use of Iodine-Impregnated Adhesive Drape

The use of iodine-impregnated adhesive drapes became standard in hip and knee arthroplasty as well as in spine surgery.

### 2.2. Statistical Analysis

The outcome variables of this study included the overall and specific surgical complication rates. Surgical complication rates were calculated using the annual surgical procedure numbers of the operating rooms.

The overall and specific complication rates between the two study periods (2015–2016 (before implementation of the complication avoidance care bundle) vs. 2017–2019 (after implementation of the bundle)) were tested for significance using the Chi-Square or 2-sided Fisher exact test. Statistical significance was set at *p* < 0.05. All statistical analysis was performed using the Statistical Package for Social Science (SPSS for Mac), version 26.0 (SPSS Inc., Chicago, IL, USA).

## 3. Results

**Figure 1:**
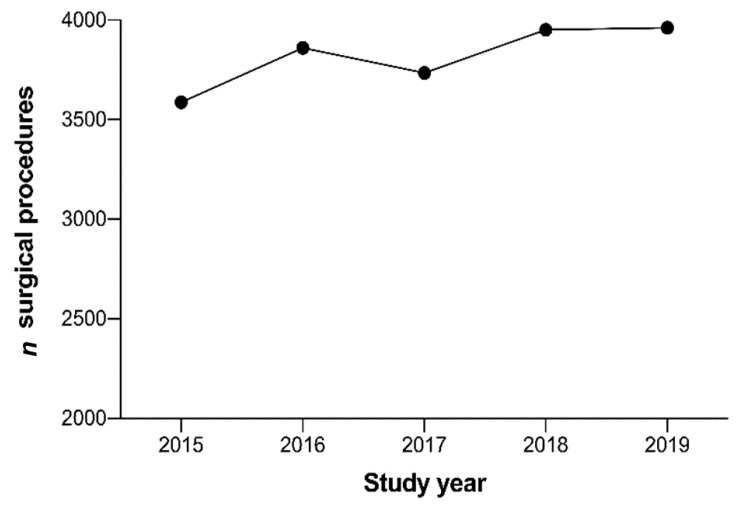
Number of surgical procedures in the study years 2015–2019.

During the study period, a total of 16,349 patients were admitted to our department and underwent surgical procedures (Table 1). The numbers of surgical procedures steadily increased over the study years, from 3587 in 2015 to 3962 in 2019 (an increase of 10.5%) (Figure 1).

**Figure 2:**
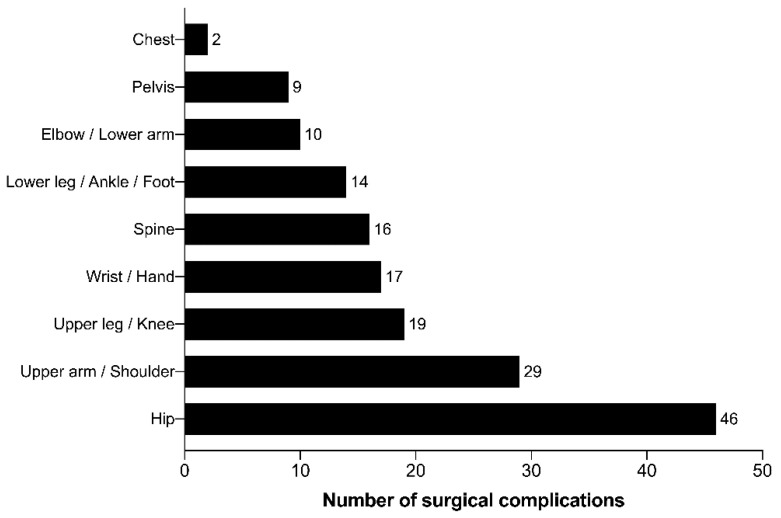
Distribution of the surgical complications among the different body areas.

Within the 5-year study period, the overall rate of surgical complications was 0.8% (overall *n* surgical complications = 161). The distribution of the anatomic region of the registered complications is shown in Figure 2. The anatomic region most often affected was the hip (*n* = 46; 28.6% of all complications), followed by the upper arm and shoulder (*n* = 29; 18.0%) and the knee (*n* = 19; 11.8%).

SSI were the complications most frequently found (*n* = 40, 24.8% of all surgical complications), followed by screw malposition (*n* = 20, 12.4%), postoperative dislocations of arthroplasties (*n* = 18, 11.2%), and suboptimal fracture reduction (*n* = 18, 11.2%).

### Complications before and after the Implementation of the “Complication Avoidance Care Bundle”

The surgical complications registered in the years 2015 and 2016 are compared to the complication rates in the years 2017–2019 in Table 1.

**Figure 3:**
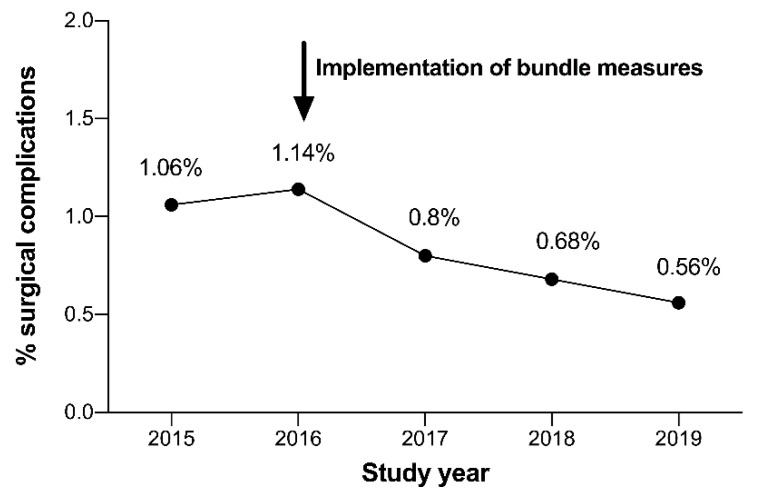
Rate of surgical complication in the study years.

The overall rate of surgical complications was 1.06% (*n* = 38) and 1.14% (*n* = 44) for the years 2015 and 2016, respectively. In January 2017, the complication avoidance care bundle was implemented in our clinic, and the previously described measures were routinely applied in all surgical procedures in our department. Thereafter, the overall rate of surgical complications continuously decreased to 0.80% (*n* = 30) in 2017, 0.68% (*n* = 27) in 2018, and 0.56% (*n* = 22) in 2019 (Figure 3) (*p* = 0.004). This represents a reduction in the overall surgical complication rate of 51% within a 3-year period.

## 4. Discussion

The present study demonstrates that an open discussion and analysis of suboptimal surgical outcomes and the creation of a bundle of specific measures to address and prevent these complications in the future can significantly improve patient care. Following implementation of a 5-measure bundle to avoid surgical complications, we were able to halve the overall complication rate within a 3-year time period. Since most of the bundle measures focused on the prevention of postoperative infections, a statistically significant decrease was observed in the rate of SSI comparing the two study periods. The specific impact the different bundle measures exert on the surgical complication rate, however, cannot be evaluated.

In our analysis, the most common surgical complications were found to be SSI. This result is not surprising since orthopedic trauma patients often present with complicated soft-tissue injuries and contaminated wounds. Open fractures and implant-related surgeries, both common situations in orthopedic trauma, are well-known risk factors for SSI. Within the observed time period, the overall rate of SSI significantly decreased from 0.32% in the years 2015/2016 and to 0.14% in the years 2017–2019. While the number of open fractures was similar between the two study periods, factors other than the implemented measures of our bundle might have contributed to this decrease in the rate of SSI. Improvements or alterations of surgical approaches or equipment could represent such confounding factors. Likewise, new implants and surgical techniques might have an impact on the rate of SSI or delayed wound healing. However, during the entire study period, no systematic changes in surgical techniques or approaches were introduced in our clinic. The effect of new osteosynthesis implants—which are sporadically implemented in likely all surgical clinics—on the complication rate, however, would need a specific analysis investigating one procedure only and cannot be addressed in an overall analysis such as ours.

Complications such as postoperative dislocation of a hip and shoulder arthroplasty, screw malposition, and periprosthetic fractures detected on postoperative X-ray or suboptimal fracture reduction are complications that are difficult to address using a preventive bundle strategy. These complications are more likely associated with the surgeon’s experience and expertise in performing the operation, as well as the difficulty of the specific procedure itself. Furthermore, the surgeon’s skills are likewise correlated with the time required for performing the surgery—the longer the duration, the higher the risk for SSI [19,20,21]. Nevertheless, by systematically analyzing possible reasons for adverse outcomes, case by case, technical aspects can be discussed during an M&M conference in an intercollegial way. While the operative experience of the trauma surgeons was comparable between the two study periods, the intraoperative imaging is likewise a factor to consider. Performing correct and standardized intraoperative fluoroscopic imaging is essential to adequately assess fracture reduction and fixation. During the second study period, a new mini C-arm (Orthoscan, Ziehm Imaging GmbH, Nürnberg, Germany) was introduced in our clinic, which was mainly used for hand and wrist surgeries. However, due to the fact that all of our C-arm devices produce high-quality intraoperative imaging, we do not believe that this was associated with a significant bias in our study.

Improving the team’s situational awareness was one of the five measures of our bundle strategy to reduce surgical complications, mainly as a target to limit insufficient surgical results such as suboptimal fracture reduction, suboptimal plate or screw position, or intraoperative lesions of relevant anatomic structures. Situational awareness includes the surgeon’s perception and comprehension of the available information in the operating theatre [22]. Surgical errors are often caused by errors of judgment, incomplete understanding of complex, continuously evolving situations, and failure of vigilance and misperceptions, even though the surgeon’s technical skills are of a high standard [23,24]. However, situational awareness is thought to occur not only at the surgeon’s individual level, but also at the team level, heavily relying on teamwork and communication. In this context, the TTO can be considered an important concept to improve situational awareness at different steps of the procedure (prior to skin incision, during the procedure, and at the end of the procedure before skin closure). Therefore, performing the TTO during every surgical procedure has become an integral part of our complication avoidance bundle.

As SSI were identified as a main component of our surgical complications, several anti-infective preventive measures were developed based on the observed adverse events and were subsequently included in the bundle. It can be clearly concluded from previous studies that the majority of SSI are contracted during the time spent in the OR and that implants represent a particular risk of SSI [20]. In this context, increasing the awareness among all OR staff to reduce OR traffic and door-opening events as much as possible is targeted at limiting the levels of airborne bacteria in the OR. Door-opening events and the movement of personnel and equipment may affect the airflow pattern in the OR and the distribution of these germs. However, the role of this form of contamination in SSI is controversial and, so far, unclear [25,26]. During the second study period, clear instructions focusing on the reduction of operating-room traffic and door-opening events were outlined at every operating-room door. However, as a clear limitation of our study, we were not able to compare the number of door-opening events or the amount of operating-room traffic between the two study periods, making an interpretation of the impact of this measure on the overall complication rate and the rate and decrease of SSI in the second study period difficult.

Routine screening for urinary tract infections (UTI) was initiated in 2017 in all patients requiring elective, but also emergent, hip surgery. The reported prevalence of preoperative UTI among elderly patients undergoing total joint arthroplasty ranges from 5.1% to 36% in the literature [27,28,29]. Several studies reported higher rates of SSI after total hip arthroplasty and osteosynthesis of femoral neck fractures in patients with preoperative symptomatic UTI [30,31]. For asymptomatic bacteriuria, however, the review by Husted et al. nicely broached the issue of preoperative urine sampling and testing in total hip and hemiarthroplasty and in total knee arthroplasty [32]. The authors concluded that testing and treatment of asymptomatic urinary tract colonization before joint replacement is unnecessary, in particular due to the fact that microbial organisms cultured from urine samples are in most cases (>66%) not the same as those cultured from SSI. This was in line with our protocol: In our patients with symptomatic UTI only (clinical symptoms and positive result in urine test strip), antibiotic treatment was immediately started in the emergency department, preoperatively. Asymptomatic bacteriuria, however, was not prophylactically treated in both study periods.

The preoperative antibiotic prophylaxis is standard in all cases with insertion of artificial implants and in surgeries with expected large dissections and anticipated higher blood loss. In our clinic, the standard antibiotic agent used for prophylaxis in any type of procedure with implantation of internal fixation devices (e.g., nails, screws, plates, wires, prosthesis) is the 2nd-generation cephalosporin Cefuroxime, with a redosing interval of 4 h. However, following analysis of the pathogen pattern found in our SSI and our local antimicrobial susceptibility profiles, a preoperative double-antibiotic prophylaxis (adding Metronidazol to Cefuroxime) was implemented for surgeries in high-risk anatomic regions in the second study period. Incision sites close to the groin and axilla, with their known high density of bacterial cells, were considered high-risk areas. Metronidazol, as an additional prophylactic antibiotic agent, therefore covers anaerobic pathogens, which were frequently isolated in the setting of SSI following surgery in these anatomic regions.

The use of iodine-impregnated adhesive drapes, in particular in hip and knee arthroplasty as well as in spine surgery, has been included as a further bundle measure focusing on the prevention of postoperative wound infections. These drapes show a microbiocidal effect in vitro. They act by sealing the skin to prevent the remaining skin flora from contaminating the surgical wound, and furthermore, the iodine may penetrate the hair follicles, killing pathogens that were not reached by the skin antiseptic [33]. Although a recently performed Cochrane analysis, comparing iodine-impregnated incision drape vs. no incision drape, was not able to demonstrate significant differences in the rate of SSI in two studies with 1113 patients [34], other authors were able to show positive effects of iodine-impregnated drapes. In the field of hip endoprosthesis implantation, no significant influence was found after the use of antiseptic drapes (*n* = 649) as compared to the use of skin antiseptic alone with PVP iodine [35]. However, it should be noted that an adequate sample size, required to confirm the efficacy of iodine-impregnated incision drapes, should be much higher due to the low rate of SSI in clean and clean-contaminated surgery [36]. Therefore, a sufficiently powered randomized controlled trial using a standardized skin preparation in arthroplasty patients is certainly needed to evaluate the impact of iodine-impregnated drapes on SSI [32].

The present study has several limitations. Due to the fact that all measures of the complication avoidance care bundle were introduced simultaneously, the effect of one specific measure on the overall or specific complication rate cannot be evaluated. This would need a prospective approach with a staged introduction of the different measures. Furthermore, we were not able to compare the amount of operating-room traffic or the number of door-opening events between the two study periods. Most of the bundle measures focused on the prevention of SSI, resulting in a significant decrease of the infection rate in the second study period. For the other specific complication rates, which were mainly approached by increasing situational awareness and introducing a structured TTO, we were not able to demonstrate a statistically significant decrease. For these more rare complications, a more detailed analysis of specific types of procedures and ideally a prospective multicenter approach would be needed. Finally, comparison of complication rates among different studies is difficult, mainly due to the fact that varying patient cohorts or different methods to identify and weigh complications are used.

## 5. Conclusions

Our multimodal strategy, targeting the reduction of surgical complication rates, was associated with a high impact and was able to reduce the complication rate by 51% within a 3-year time period. The combination of these five interventions was established based on the surgical complications noted in our department and subsequent transparent discussion in a weekly M&M conference. In addition to including systemic changes, such as routine screening for infective foci in patients requiring hip surgery and adapting the preoperative antibiotic prophylaxis in high-risk anatomic regions, these measures likewise focused on improving teamwork, communication, and attitudes towards quality and patient safety.

## Figures and Tables

**Table 1:** Surgical complications stratified by study years.

	All Years	2015–2016	2017–2019	*p*-Value
Patients admitted to hospital	16,349	6677	9672	
Total number of surgical procedures	19,096	7448	11,648	
All surgical complications, % (*n*)	0.84% (161)	1.10% (82)	0.68% (79)	0.002
Surgical site infection (SSI), % (*n*)	0.21% (40)	0.32% (24)	0.14% (16)	0.006
Early postoperative dislocation after arthroplasty, % (*n*) *	0.09% (18)	0.11% (8)	0.09% (10)	0.636
Periprosthetic fracture, % (*n*)	0.05% (10)	0.08% (6)	0.03% (4)	0.173
Screw malposition, % (*n*) **	0.1% (20)	0.15% (11)	0.08% (9)	0.142
Suboptimal fracture reduction, % (*n*)	0.09% (18)	0.13% (10)	0.07% (8)	0.150
Postoperative neurological deficit, % (*n*) ***	0.06% (12)	0.09% (7)	0.04% (5)	0.170
Intraoperative bleeding complication, % (*n*)	0.05% (9)	0.07% (5)	0.03% (4)	0.309
Various, % (*n*)	0.18% (34)	0.15% (11)	0.2% (23)	0.426

* Dislocation of reversed shoulder arthroplasty and hip arthroplasty, detected on postoperative X-ray. ** Intra-articular or intraspinal screw placement, detected on postoperative X-ray. *** Postoperative peripheral neurological deficit, including radial/ulnar/femoral/ischiadic/peroneal nerve palsy.
